# Impact of 5–20% Hydroponic Wheat Sprouts Inclusion on Growth and Metabolic Parameters of Growing Ewes

**DOI:** 10.3390/ani14111630

**Published:** 2024-05-30

**Authors:** Li Min, Yong Tuo, Dagang Li, Changjiang Zang, Guzalnur Amat, Zhijun Zhang, Tongjun Guo

**Affiliations:** 1 Key Laboratory of Xinjiang Feed Biotechnology, Feed Research Institute, Xinjiang Academy of Animal Science, Urumqi 830011, China; minli@gdaas.cn (L.M.);; 2 Ministry of Agriculture Key Laboratory of Animal Nutrition and Feed Science in South China, Institute of Animal Science, Guangdong Academy of Agricultural Sciences, Guangzhou 510640, China; lidagang@gdaas.cn; 3 College of Animal Science, Xinjiang Agricultural University, Urumqi 830052, China; zcj780@126.com

**Keywords:** unconventional feeds, alternative forages, economic effectiveness, rumen manipulation, growing ewes

## Abstract

**Simple Summary:**

Simple Summary: Hydroponic wheat sprouts could be used as a potential sustainable feedstuff resource for livestock production. Based on its nutrient composition, it could exert good palatability and digestibility, thereby improving the feed intake and productive performance of livestock. We have evaluated the responses of four-month-old growing Hu ewes, while their diets are replaced with different proportions (5–20%) of hydroponic wheat sprouts. In this experiment, substitution 15% of the basal diet with hydroponic wheat sprouts would obtain the optimum production performance and net profit for growing ewes. Inversely, the higher substitution ratio (20%) will result in the decline of production performance and economic effectiveness, and show negative effect on rumen fermentation.

**Abstract:**

The aim of this study was to assess the impact of varying proportions (5–20%) of hydroponic wheat sprouts in the diet of growing four-month-old Hu ewes on their productive performance, metabolic profiles, rumen fermentation, and alterations in microflora. Compared with the control group (CON), the optimum final weight of ewes has been presented in the group of substitution 15% (S15) of the basal diet with hydroponic wheat sprouts. Furthermore, 1–30 d the average daily gain (ADG), 31–60 d ADG, and average feed intake were both significantly improved in S15 compared with CON (*p* < 0.05). Feeding hydroponic wheat sprouts can significantly increase high-density lipoprotein and interleukin-2 (*p* < 0.05) accompanied by the numerical increase of the content of interferon-γ, suggesting its positive effect on ewes’ health and immune systems. In this process, it is noteworthy that feeding hydroponic wheat sprouts results in an increase in relative abundance of *Olsenella*, *Limosilactobacillus*, *Shuttleworthia*, and *Prevotella*_7, and a decrease in relative abundance of *Succinimonas*, *Pseudobutyrivibrio*, and *Anaerovibrio* in the rumen of growing ewes. It implies that the response of rumen microflora adapted to the change of dietary ingredients, as well as the relationship between rumen microflora changes and the improvement of productive performance and immune system in growing ewes. Considering the usage cost and application effect, S15 of the basal diet with hydroponic wheat sprouts could be the appropriate application solution for growing ewes.

## 1. Introduction

The growing population coupled with the decreasing arable land worldwide have intensified the challenge of sustainable food supply chains [1]. Currently, hydroponic fodder has been used as a potential sustainable feedstuff resource for livestock production, which could be beneficial to reducing the food-feed competition and even improving productive performance [2]. Furthermore, owing to the rising prices of protein and energy feedstuff, feeding hydroponic fodder has also been used as an alternative solution extensively worldwide to reduce feed costs [3]. Therein, sprouted wheat is wheat grain that has been soaked in water, placed in trays, and allowed to germinate and ultimately convert into green feed within 6 to 8 days [4]. It requires soilless culture, less water and labor, and enables production at any time of the year. More importantly, compared with wheat grain, hydroponic wheat sprouts can provide more biomass, and also provide high-quality roughage for ruminants, which would be beneficial to rumen function. Ma et al. [5] indicated that hydroponic wheat sprouts are rich in protein, multi-amino acids, vitamins, and other nutritional components, which could exert good palatability and digestibility, thereby improving the feed intake and productive performance of livestock. However, limited research is available on the application of feeding hydroponic wheat sprouts on growing ewes.

To date, the application of hydroponic fodder is mainly focused on hydroponic barley sprouts in sheep production. Feeding hydroponic barley sprouts to sheep could result in better digestibility and rumen fermentation, as well as high animal performance [2]. Supplements of sprouted barley at different levels can improve the digestibility and rumen fermentation characteristics of Awassi lambs [6]. Feeding hydroponic barley sprouts could also be beneficial to lactating ewes, and replacing 5–15% of the basal diet could alleviate the oxidative stress of lactating ewes and improve the milk quality [7].

As regards to hydroponic wheat sprouts, Guerrero-Cervantes, et al. [8] found that hydroponic wheat sprouts are a suitable solution for alternative corn and cottonseed meals in the diets of Katahdin ewes (up to 30%) during gestation and lactation without having a negative effect, but instead having a beneficial effect on their reproductive performance, indicated by the growth and development of their lambs. Currently, little information is available about hydroponic wheat sprout food on productive performance, metabolic profiles, rumen fermentation, and the microflora changes of growing ewes. Therefore, the objective of this study was to evaluate the responses of four-month-old growing Hu ewes, while their diets are replaced with different proportions (5–20%) of hydroponic wheat sprouts.

## 2. Materials and Methods

### 2.1. Animals and Experimental Design

All animal procedures performed in this study were approved and guided by the Animal Care and Use Committee of the Xinjiang Academy of Animal Sciences (FRI-2023002, Urumqi, China). Fifty healthy four-month-old growing Hu ewes with a homogeneous genetic background and similar body weight (30.83 ± 0.52 kg) were selected for the experiment. All of the ewes were fed the same diet to maintain the consistency of the rumen function before the experiment. The experiment was conducted under the same feeding and management conditions. They were randomly divided into five groups in a randomized complete block design, each consisting of 10 ewes, and fed with different total mixed ration diets. The nutrient requirements of growing ewes were formulated according to feeding standards for meat-producing sheep and goats in China (NY/T 816-2021 [9]). The control group (CON) was fed with the basal diet including: corn straw, alfalfa, corn, cottonseed meal, wheat bran, calcium carbonate, sodium bicarbonate, salt, and premix. As we measured in the present study, the dry matter content of hydroponic wheat sprouts is 16.94%, the crude protein content is 15.36%, the crude fat content is 4.60%, the neutral detergent fiber is 30.12%, the acid detergent fiber is 18.40%, the calcium content is 0.49%, and the phosphorus content is 0.79%. The diets of the treatment groups were used in an equal replacement method by substitution 5% (S5), 10% (S10), 15% (S15) and 20% (S20) of the basal diet (dry matter basis) with hydroponic wheat sprouts, respectively (Table 1). Briefly, hydroponic wheat sprouts can partially replace the expensive protein feed and roughage. The application of hydroponic wheat sprouts may reduce feed cost and the food-feed competition (owing to having more biomass than wheat grain), and also may improve productive performance.

### 2.2. Production Data and Sample Collection

The experiment lasted two months. Ewes in each group were housed in individual units with ad libitum access to corresponding diets. The amount of feedstuff offered and refused was weighed daily, thus calculating the feed intake. At the beginning (0 d), mid-term (30 d), and end (60 d) of the experiment, ewes were weighed after fasting for 12 h. At the end of the experiment, blood samples were collected, and then serum samples were obtained via jugular venipuncture and centrifuged at 3000× *g* for 15 min (4 °C). Meanwhile, five ewes were randomly selected from each group, and rumen fluid samples were collected via esophageal tubing and filtered through four layers of sterilized cheesecloth at the end of the experiment after fasting for 12 h. The pH value was measured immediately by pH meter, and the remaining parts were stored at −80 °C for the determination of rumen fermentation and microflora changes.

### 2.3. Serum Biochemistry Analysis

The concentrations of total protein, albumin, glucose, blood urea nitrogen, triglyceride, total cholesterol, high-density lipoprotein, low-density lipoprotein, superoxide dismutase, glutathion peroxidase, malonaldehyde, lactic dehydrogenase, creatine kinase, glutamic pyruvic transaminase, glutamic oxalacetic transaminase, interleukin-1β, interleukin-2, interferon-γ, and CD4+ in serum were determined and analyzed by Thermo Scientific Microplate Reader using commercially available enzyme-linked immunosorbent assay kits (Nanjing Jiancheng Biological Engineering Institute, Nanjing, China).

### 2.4. Rumen Fermentation Characteristics Determination

After thawing, ruminal supernatant was obtained by centrifugation (1000× *g* at 4 °C for 10 min) for the analysis of volatile fatty acids. The concentrations of volatile fatty acids in ruminal samples were analyzed as described by Mohammed, et al. [10] using a gas chromatograph (GC-2010 Shimadzu, Kyoto, Japan). The concentration of ammoniacal nitrogen was detected based on alkaline sodium hypochlorite-phenol spectrophotometry [11].

### 2.5. Rumen Microflora Sequencing and Analysis

Microbial DNA was extracted from rumen fluid samples using cetyltrimethylammonium bromide method accompanied by bead beating [12]. The bacterial 16S rRNA V3-V4 region was amplified using referenced primers 338F (ACTCCTACGGGAGGCAGCA) and 806R (GGACTACHVGGGTWTCTAAT) [13]. Purified amplicons were pooled in equimolar amounts and paired-end sequenced on an Illumina PE300 platform (Illumina, San Diego, CA, USA) according to the standard protocols. The raw sequencing reads were deposited into the NCBI Sequence Read Archive (SRA) data: PRJNA1076690.

Bioinformatic analysis of the rumen microflora was conducted by the BMK cloud platform (http://www.biocloud.net/ accessed on 30 January 2024). According to the quality of a single nucleotide, raw data were primarily filtered by Trimmomatic (version 0.33). Identification and removal of primer sequences was processed by Cutadapt (version 1.9.1). PE reads obtained from previous steps were assembled by USEARCH (version 10) and followed by chimera removal using UCHIME (version 8.1). Sequences with similarity >97% were clustered into the same operational taxonomic unit (OTU) by USEARCH (v10), and the OTUs counts less than 2 in all samples were filtered.

The qualified sequences with more than 97% similarity thresholds were allocated to one OTU using USEARCH (version 10.0). Taxonomy annotation of the OTUs was performed based on the Naive Bayes classifier in QIIME2 using the SILVA database (release 138.1) with a confidence threshold of 70%. Alpha and beta diversity were calculated and displayed by the QIIME2. One-way analysis of variance was used to compare bacterial abundance and diversity. Linear discriminant analysis (LDA) coupled with effect size (LEfSe) was applied to evaluate the differentially abundant taxa (LDA score > 2, *p* < 0.05).

### 2.6. Statistical Analysis

Statistical analysis was performed using SPSS 23.0 statistical program. All experiment data were tested and confirmed for normal distribution using the Shapiro-Wilk test. Subsequently, the data of productive performance, serum biochemistry, and rumen fermentation characteristics were analyzed statistically using the one-way ANOVA procedure accompanied by Tukey’s test. The results of the test were displayed as the mean and standard error of the mean (SEM), and *p* < 0.05 as significant level of difference.

## 3. Results

### 3.1. Effect of Feeding Hydroponic Wheat Sprouts on Productive Performance and Economic Effectiveness of Growing Ewes

Although there was no significant difference in final weight of ewes among the diet of five groups, the optimum final weight of ewes has been presented in S15, with the increased of average 3.14 kg compared with CON (Table 2). Furthermore, 1–30 d ADG, 31–60 d ADG, and average feed intake were both significantly improved (*p* < 0.05) in S15 compared with CON. Finally, the feed to gain ratio is 10.07, 10.15, 9.02, 8.87, and 9.76 from the groups of CON to S5, S10, S15, and S20 in turn.

The other important purpose of the application of feeding hydroponic wheat sprouts is to reduce feeding costs. As shown in Table 3, the minimum of feed unit price and the maximum net profit have been obtained in S15 according to the current market price in Kashgar, Xinjiang, China. Furthermore, if the price of hydroponic wheat sprouts is increased to more than 0.8 CNY/kg in future, it is inappropriate to use hydroponic wheat sprouts as a feedstuff resource to partially replace the basal diet for livestock production (Table 3).

### 3.2. Effect of Feeding Hydroponic Wheat Sprouts on Serum Metabolic Profiles of Growing Ewes

Most blood biochemical indexes do not significantly change after feeding different proportions of hydroponic wheat sprouts to growing ewes (Table 4). It is noteworthy that feeding hydroponic wheat sprouts causes a varying degree of increase (*p* < 0.05) in glutamic oxalacetic transaminase, urea nitrogen, high-density lipoprotein, and interleukin-2 in growing ewes.

### 3.3. Effect of Feeding Hydroponic Wheat Sprouts on Rumen Fermentation and Microflora Changes of Growing Ewes

S20 of the basal diet with hydroponic wheat sprouts exerted the lowest value of rumen fermentation parameters in growing ewes among the diets of five groups, including ammoniacal nitrogen, total volatile fatty acids, acetate, and valerate (Table 5). In addition to S20, there was no significant difference in the results of ammoniacal nitrogen and volatile fatty acids among other groups.

**Figure 1:**
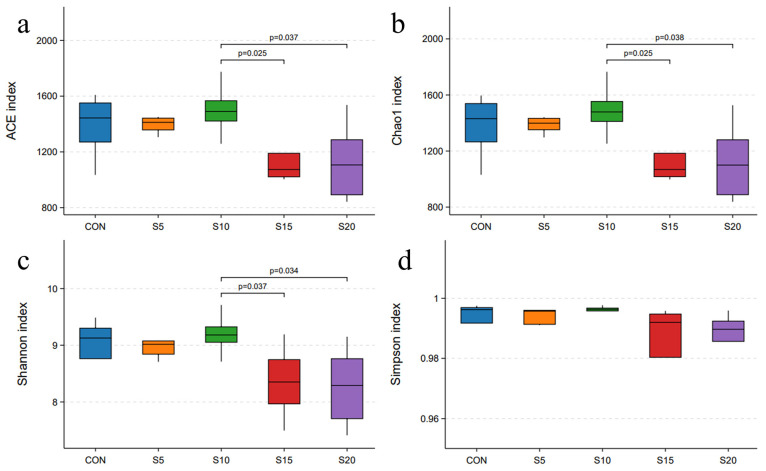
Effect of feeding hydroponic wheat sprouts on the alpha diversity index of rumen bacteria in growing ewes. (**a**): ACE index, (**b**): Chao 1 index, (**c**): Shannon index, (**d**): Simpson index.

**Figure 2:**
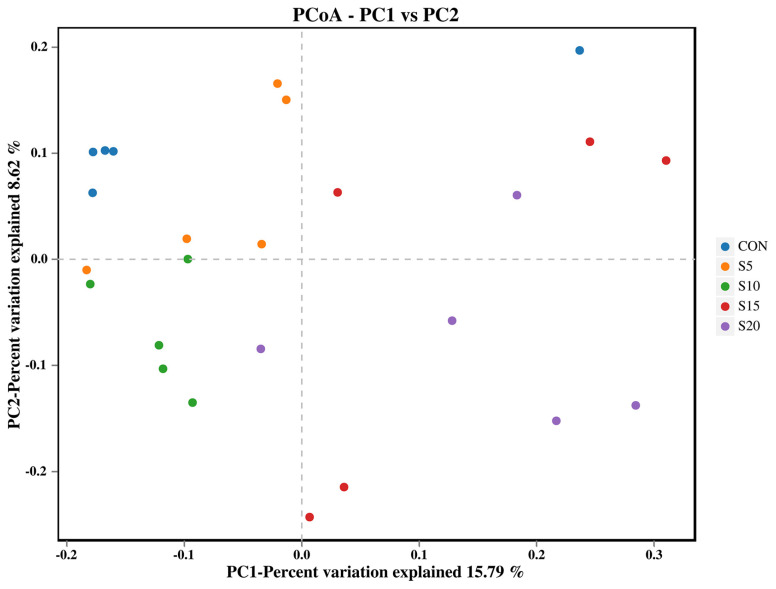
Principal coordinate analysis of the effect of feeding hydroponic wheat sprouts on the rumen bacterial community based on the unweighted unifrac distance.

**Figure 3:**
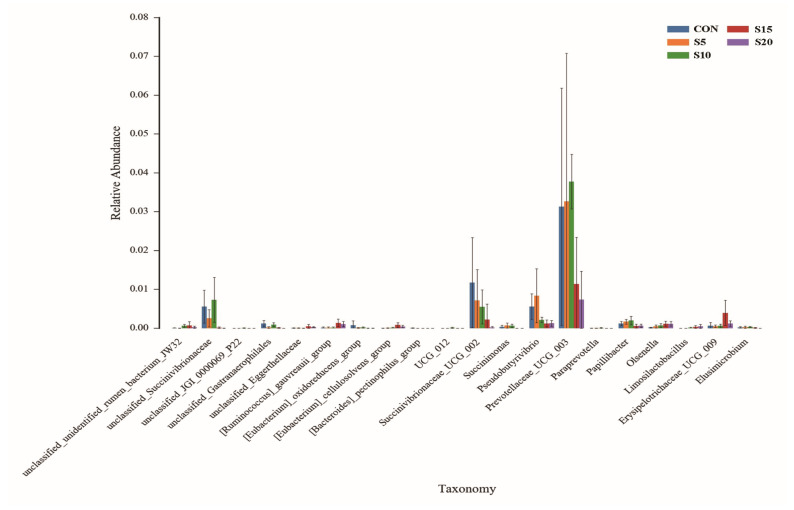
Effect of feeding hydroponic wheat sprouts on the microflora changes of growing ewes in the rumen bacterial community using the Wilcoxon rank-sum test.

**Figure 4:**
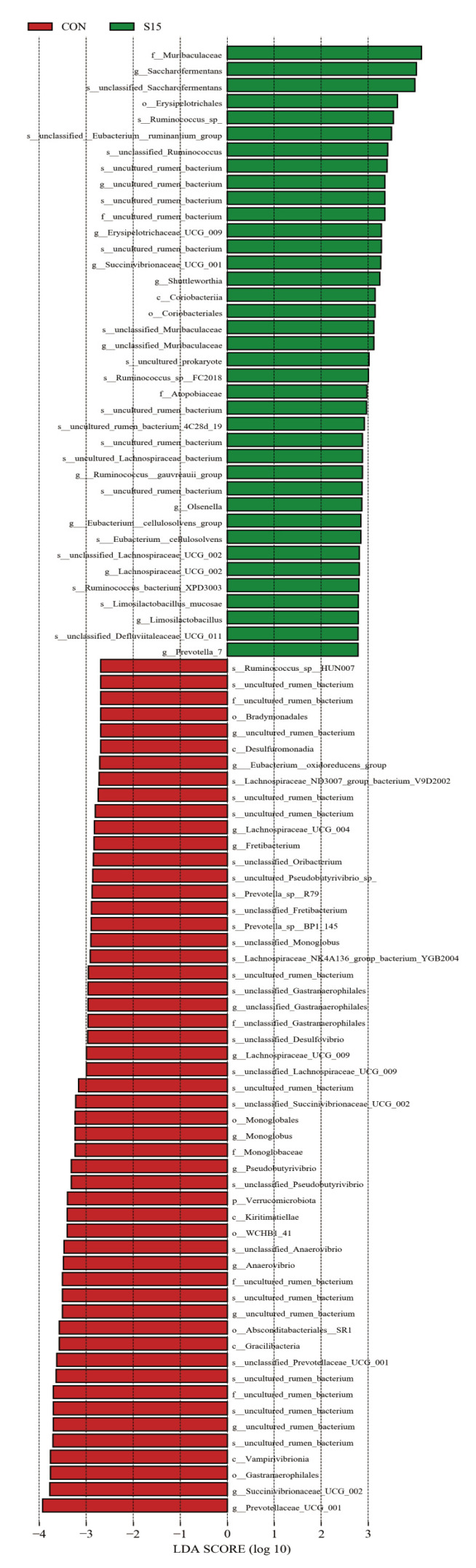
Linear discriminant analysis effect size (LEfSe) analysis of the microflora changes of growing ewes in the rumen bacterial community by substitution 15% (S15) of the basal diet with hydroponic wheat sprouts.

With respect to microflora changes, the alpha diversity analysis, beta diversity analysis, and rumen microorganism composition changes were presented in Figure 1, Figure 2, Figure 3 and Figure 4. S15 and S20 of the basal diet with hydroponic wheat sprouts resulted in a decrease in the richness indexes of ACE (Figure 1a) and Chao 1 index (Figure 1b), and also diversity index of Shannon index (Figure 1c). No significant difference was observed in the Simpson index (Figure 1d).

Principal coordinate analysis (PCoA) based on the unweighted unifrac distance was performed to compare the bacterial community (Figure 2). There is a relatively clear separation among the diets of five groups.

Based on the Wilcoxon rank-sum test, we found that with the increasing of substitution with hydroponic wheat sprouts, there were obviously increases in the relative abundance of [*Ruminococcus*]_gauvreauii_group, *Olsenella*, *Limosilactobacillus*, and Erysipelotrichaceae_UCG_009 (Figure 3). Meanwhile, there were obviously decreases in the relative abundance of unclassified_Succinivibrionaceae, Succinivibrionaceae_UCG_002, *Succinimonas*, *Pseudobutyrivibrio*, Prevotellaceae_UCG_003, and *Papillibacter* (Figure 3).

Furthermore, LEfSe analysis revealed that compared with CON, S15 of the basal diets with hydroponic wheat sprouts have given rise to a significant increase in the relative abundance of *Saccharofermentans*, Erysipelotrichaceae_UCG_009, Succinivibrionaceae_UCG_001, *Shuttleworthia*, unclassified_Muribaculaceae, *Ruminococcus*__gauvreauii_group, *Olsenella*, *Eubacterium*__cellulosolvens_group, Lachnospiraceae_UCG_002, and *Limosilactobacillus*, Prevotella_7 (Figure 4). Simultaneously, there was a reduction in the relative abundance of *Eubacterium*__oxidoreducens_group, Lachnospiraceae_UCG_004, *Fretibacterium*, unclassified_Gastranaerophilales, Lachnospiraceae_UCG_009, *Monoglobus*, *Pseudobutyrivibrio*, *Anaerovibrio*, Succinivibrionaceae_UCG_002, and Prevotellaceae_UCG_001 (Figure 4).

## 4. Discussion

Hydroponic wheat sprouts were found to contain a high protein content and high-quality fiber, suggesting that it could be used as a high-quality roughage resource for growing ewes. In this experiment, S15 of the basal diet with hydroponic wheat sprouts would obtain the optimum production performance and net profit. Nevertheless, the higher substitution ratio (S20) will result in the decline of production performance, net profit, and even negative effect on rumen fermentation. Guerrero-Cervantes, et al. [8] indicated that hydroponic wheat sprouts are a suitable substitute for the basal diet in ewes’ diets during gestation and lactation without having a detrimental effect. However, owing to the excessive substitution ratio (30%) in this process, the replacement strategy did not improve the feed intake of the ewes during gestation and daily gain of the lambs [8]. Hence, based on the response of using different proportions of hydroponic wheat sprouts in the present study, we recommended that S15 of the basal diet with hydroponic wheat sprouts might be the appropriate application solution for growing ewes. The diet with 15% hydroponic wheat sprouts had lower fiber values, which would influence the growth performance, metabolism, and ruminal parameters of the ewes.

However, there was no significant impact on the metabolism of the growing ewes after feeding them hydroponic wheat sprouts, as reflected by most blood biochemical indexes. Intriguingly, the content of glutamic oxalacetic transaminase and urea nitrogen had a tendency to increase in all hydroponic wheat sprouts substitution groups, which might suggest the promotion of liver metabolism [7] and nitrogen metabolism [14] in growing ewes. Theoretically, the increase in serum glutamic oxalacetic transaminase might not be beneficial to health. However, glutamic oxaloacetic transaminase is involved in the catalysis in the process of ammonia transfer on wheat, soybean, corn, and other major crops [15]. It plays an important role in catalyzing the conversion of glutamate to aspartic in plants. The activity of glutamic oxaloacetic transaminase and its related metabolites in S5-S20 might be higher than CON, which might also result in the increase of serum glutamic oxalacetic transaminase in growing ewes in this study. Similar to this phenomenon, feeding hydroponic barley seedlings to lactating ewes will significantly increase the blood glutamic oxalacetic transaminase value in lambs [7].

It is well known that the role of high-density lipoprotein consists of mobilizing circulating cholesterol to the liver to be finally metabolized [16]. In this sense, the elevation of high-density lipoprotein as a result of feeding hydroponic wheat sprouts here would avoid the accumulation of cholesterol in peripheral tissues impacting positively on ewes’ health and welfare. Chang et al. [17] elucidated that wheat sprouts could significantly attenuate the accumulation of lipid droplets in liver cells and non-alcoholic fatty liver disease-induced mice through the AMPK pathway activity, accompanied by the decrease of total cholesterol in the blood. Another study indicated that the extraction of wheat sprouts could suppress acetaminophen-induced hepatotoxicity in mice, manifested by inhibiting oxidative liver damage and reducing the expression of inflammatory cytokines [18]. The observed effects on metabolic profiles in growing ewes might reveal the hepatoprotective effect of feeding hydroponic wheat sprouts.

Interleukin-2 is a key cytokine with broad immunomodulatory activity. It could prevent the uncontrolled expansion of immune responses and limit overall inflammation [19]. Additionally, interleukin-2 induces interferon-γ production, which is a cytokine that also plays a vital role in inducing and modulating immune systems [20]. As shown in Table 4, feeding hydroponic wheat sprouts can numerically increase the content of interferon-γ accompanied by the significantly increase of interleukin-2. In the present study, the increase in interleukin-2 and interferon-γ suggests that feeding hydroponic wheat sprouts might improve immune systems in growing ewes.

The microflora changes of growing ewes in this study also supports the idea that feeding them hydroponic wheat sprouts might improve their immune systems. It was found that an increase in the abundance of *Olsenella* was considered to improve immunity in calves [21]. Moreover, *Olsenella* was also considered to strengthen the immunoregulation capacity of a goat model [22] and mouse model [23] host. Similarly, *Limosilactobacillus* has been proved to have antibacterial and intestinal immunity-enhancing effects [24]. An increase in the abundance of *Limosilactobacillus* would facilitate the expression of colon immune factors in rats, which indicated that the gut flora changes of *Limosilactobacillus* can also improve the body’s immune system [25]. Taken together, the increase in the abundance of *Olsenella* and *Limosilactobacillus* (Figure 3 and Figure 4) after feeding hydroponic wheat sprouts might show an improvement in the immune systems of growing ewes as demonstrated by the increase of interferon-γ and interleukin-2 (Table 4).

Additionally, the change of diet structure by replacement with hydroponic wheat sprouts is likely to lead to the change of rumen microflora in growing ewes. As shown in Table 1, with an increasing substitution of hydroponic wheat sprouts (S15), there is a reduction of usage amount of corn straw and a decrease in the nutrient composition of neutral detergent fiber and acid detergent fiber. *Pseudobutyrivibrio* is an important member of the rumen microbiome contributing to the degradation and utilization of lignocellulosic plant material [26]. Li et al. [27] demonstrated that the abundance of *Pseudobutyrivibrio* has increased linearly with increasing neutral detergent fiber and acid detergent fiber in the diet of sheep. In addition to *Pseudobutyrivibrio*, Ma et al. [28] indicated that *Succinimonas* in the rumen also play a critical role in forage degradation. Conversely, owing to the reduction in the usage of corn straw (containing more xylan, hemicellulose, and fiber) in this study, there were decreases in the abundance of *Pseudobutyrivibrio* and *Succinimonas*, suggesting the rumen microflora adapted to the change in dietary ingredients. Analogously, it is worth noting that the increase in the abundance of *Shuttleworthia* and *Prevotella*_7 occurred in the hydroponic wheat sprouts-fed rumen microbiome, which is similar to what happened in a previous study. *Shuttleworthia* and *Prevotella*_7 genera were significantly enhanced in the wheat-fed rumen microbiome [29].

The improvement of the productive performance in growing ewes might also be related to rumen microflora changes. There was a greater relative abundance of *Shuttleworthia* in the rumen of low-residual feed intake (high-efficiency) heifers [30] and dairy cows [31]. Moreover, Kim [32] has summarized that *Anaerovibrio* in the rumen was less abundant in the high-efficiency group of ruminants. It was also found that the presence of Succinivibrionaceae_UCG_002 was inversely proportional to feed efficiency [33]. Therefore, the increase in the abundance of *Shuttleworthia* and decrease in the abundance of *Anaerovibrio* and Succinivibrionaceae_UCG_002 in the rumen could support the results of the improvement of final weight, ADG, feed intake, and feed-to-gain ratio in the present study. However, the underlying mechanism during this process still needs further research.

## 5. Conclusions

In this experiment, S15 of the basal diet with hydroponic wheat sprouts would obtain an optimum production performance and net profit for growing ewes. However, the higher substitution ratio (20%) will result in the decline of production performance and economic effectiveness, and have a negative effect on rumen fermentation. Feeding hydroponic wheat sprouts might improve immune system and feed efficiency in growing ewes, indicated by the blood biochemical indexes and rumen microflora changes. Due to the function of these rumen microflora, the increase in the abundance of *Olsenella* and *Limosilactobacillus* after feeding hydroponic wheat sprouts might evidence the improvement of immune systems in growing ewes. Furthermore, the increase in the abundance of *Shuttleworthia* and decrease in the abundance of *Anaerovibrio* and Succinivibrionaceae_UCG_002 in the rumen could support the results of the improvement of feed efficiency. With the reducing usage of corn straw, there were decreases in the abundance of the rumen microbiome (*Pseudobutyrivibrio* and *Succinimonas*) contributing to the degradation and utilization of lignocellulosic plant material. Overall, we recommended that S15 of the basal diet with hydroponic wheat sprouts could be the appropriate application solution for growing ewes. The application of hydroponic wheat sprouts (S15) is likely to improve productive performance, reduce feeding costs, and alleviate the food competition between humans and animals. It could be demonstrated and promoted in Xinjiang and throughout the country for sheep production.

## Figures and Tables

**Table 1:** Dietary ingredients and nutrient composition (dry matter basis) for four-month-old growing Hu ewes in this experiment.

Item	Groups				
	CON	S5	S10	S15	S20
Ingredients (%)					
Hydroponic wheat sprouts	0	5	10	15	20
Corn straw	32.0	31.5	29.0	26.0	24.5
Alfalfa	10	7	6	5	3
Corn	30.0	29.0	28.8	28.8	28.0
Cottonseed meal	19.5	18.9	17.59	16.4	15.6
Wheat bran	4	4	4	4	4
Calcium carbonate	0	0.1	0.2	0.3	0.4
Sodium bicarbonate	0.7	0.7	0.7	0.7	0.7
Salt	0.8	0.8	0.8	0.8	0.8
Premix ^1^	3	3	3	3	3
Nutrient composition (%)					
Dry matter content	87.09	83.59	80.39	77.06	73.68
Crude protein	16.01	15.93	15.92	16.08	16.09
Crude fat	2.69	2.91	3.18	3.41	3.55
Neutral detergent fiber	27.90	27.77	26.88	23.00	23.47
Acid detergent fiber	16.30	14.72	14.68	11.58	11.00
Ash	11.59	12.18	11.78	11.77	11.38
Calcium	0.84	0.85	0.88	0.91	0.93
Phosphorus	0.53	0.55	0.57	0.59	0.61
Metabolic energy (MJ/kg)	11.19	11.19	11.19	11.19	11.19

^1^ Premix contained the following per kg of diet: VA 1,000,000 IU, VD3 65,000 IU, VE 5000 mg, Fe 2000 mg, Cu 1750 mg, Zn 5500 mg, Mn 2550 mg, Se 75 mg, I 70 mg, Co 40 mg.

**Table 2:** Effect of feeding hydroponic wheat sprouts on productive performance of growing ewes.

Item	Groups					SEM	*p* Value
	CON	S5	S10	S15	S20		
Initial weight (kg)	30.53	30.54	30.96	30.93	31.18	0.52	0.99
Final weight (kg)	36.96	37.89	38.50	40.10	39.39	0.56	0.44
1–30 d ADG (g/d)	115.95 ^b^	121.67 ^b^	134.52 ^b^	165.95 ^a^	137.38 ^b^	5.00	<0.01
31–60 d ADG (g/d)	93.06 ^b^	105.56 ^ab^	120.28 ^ab^	139.05 ^a^	130.55 ^a^	5.16	0.02
ADG (g/d)	106.25	111.31	128.57	145.42	136.31	6.74	0.32
Average feed intake (kg/d)	1.07 ^c^	1.13 ^bc^	1.16 ^b^	1.29 ^a^	1.33 ^a^	0.01	<0.01
Feed to gain ratio	10.07	10.15	9.02	8.87	9.76		

ADG: average daily gain. Within rows, means with different letter superscripts (^a–c^) are significantly different (*p* < 0.05).

**Table 3:** Economic effectiveness analysis of the application of feeding hydroponic wheat sprouts in growing ewes.

Item	Groups				
	CON	S5	S10	S15	S20
Total weight gain (kg/piece)	6.43	7.35	7.54	9.17	8.21
Feed unit price (CNY/kg) ^1^	2.32	1.89	1.61	1.42	1.27
Total feed intake (kg/piece)	71.01	93.28	121.41	150.08	177.23
Feed cost (CNY/piece)	164.78	176.15	195.64	212.51	224.27
Weight gain profit (CNY/piece) ^2^	210.38	220.39	254.57	287.93	269.89
Net profit (CNY/piece)	45.59	44.24	58.93	75.42	45.63
The price change of hydroponic wheat sprouts ^3^					
0.5 CNY/kg	45.59	44.24	58.93	75.42	45.63
0.6 CNY/kg	45.59	41.99	54.06	67.67	34.96
0.7 CNY/kg	45.59	39.74	49.18	59.92	24.29
0.8 CNY/kg	45.59	37.48	44.30	52.17	13.62
0.9 CNY/kg	45.59	35.23	39.42	44.42	2.95

CNY: China Yuan. ^1^ The price of feed raw materials is calculated according to the current market price in Kashgar, Xinjiang (May 2022), which is used in the present study as follows: hydroponic wheat sprouts 0.5 CNY/kg, corn straw 0.85 CNY/kg, alfalfa 1.8 CNY/kg, corn 2.65 CNY/kg, cottonseed meal 3.75 CNY/kg, wheat bran 2.8 CNY/kg, calcium carbonate 2.5 CNY/kg, sodium bicarbonate 2.8 CNY/kg, salt 0.85 CNY/kg, premix 5.8 CNY/kg. ^2^ Unit price of live weight of Hu sheep is calculated according to the current market price in Kashgar, Xinjiang (August 2022): 33 CNY/kg. ^3^ If the price of hydroponic wheat sprouts is increased from 0.5 CNY/kg to 0.9 CNY/kg, the net profit of the application of feeding hydroponic wheat sprouts in growing ewes is presented among each group.

**Table 4:** Effect of feeding hydroponic wheat sprouts on serum metabolic profiles of growing ewes.

Item	Groups					SEM	*p* Value
	CON	S5	S10	S15	S20		
Total protein (g/L)	53.21	61.16	64.60	61.39	59.81	1.42	0.13
Albumin (g/L)	13.66	16.16	16.42	13.85	15.56	0.40	0.07
Superoxide mutase (U/mL)	156.79	166.43	162.27	154.78	149.56	5.06	0.88
Malonaldehyde (nmol/mL)	3.50	3.36	3.03	2.74	2.97	0.11	0.12
Glutathione peroxidase (U/mL)	22.44	24.87	23.43	24.49	25.92	0.97	0.83
Lactate dehydrogenase (U/L)	3333.3	3586.2	3697.9	3348.7	3738.5	75.2	0.26
Creatine kinase (U/L)	0.101	0.091	0.110	0.124	0.101	0.006	0.51
Alanine aminotransferase (U/L)	2.22	2.64	2.56	2.81	2.40	0.09	0.27
Glutamic oxalacetic transaminase (U/L)	8.04 ^b^	10.63 ^a^	11.35 ^a^	10.40 ^ab^	11.03 ^a^	0.32	<0.01
Urea nitrogen (mmol/L)	5.80 ^b^	6.60 ^a^	7.30 ^a^	6.12 ^b^	6.41 ^ab^	0.14	0.01
Glucose (mmol/L)	2.14	2.25	2.71	2.24	2.6	0.10	0.28
Triglyceride (mmol/L)	0.26	0.31	0.34	0.25	0.27	0.02	0.52
Total cholesterol (mmol/L)	1.08	1.23	1.35	1.18	1.21	0.05	0.52
High-density lipoprotein (mmol/L)	0.35 ^c^	0.41 ^bc^	0.54 ^a^	0.51 ^ab^	0.54 ^a^	0.02	<0.01
Low-density lipoprotein (mmol/L)	0.39	0.43	0.48	0.4	0.43	0.02	0.59
Interleukin-1β (pg/mL)	25.47	28.47	29.24	26.56	29.2	0.57	0.12
Interleukin-2 (pg/mL)	28.91 ^b^	34.33 ^ab^	35.83 ^a^	34.96 ^a^	34.65 ^a^	0.7	0.01
Interferon-γ (pg/mL)	65.02	67.00	73.60	72.21	74.61	1.82	0.38
CD4+ (ng/mL)	203.8	236	263.9	236.1	229.6	7.20	0.14

Within rows, means with different letter superscripts (^a–c^) are significantly different (*p* < 0.05).

**Table 5:** Effect of feeding hydroponic wheat sprouts on rumen fermentation parameters of growing ewes.

Item	Groups					SEM	*p* Value
	CON	S5	S10	S15	S20		
pH	6.17	6.14	6.24	6.22	6.11	0.02	0.16
Ammoniacal nitrogen (mol/100 mL)	23.30	24.43	23.10	26.10	19.33	0.83	0.12
Acetate (mmol/L)	57.62	69.52	56.39	60.53	54.95	2.01	0.07
Propionate (mmol/L)	17.47	18.38	14.52	18.33	15.77	0.65	0.24
Butyrate (mmol/L)	12.10	14.15	12.05	12.73	12.22	0.45	0.56
Valerate (mmol/L)	2.28 ^ab^	2.01 ^ab^	2.09 ^ab^	2.48 ^a^	1.92 ^b^	0.06	0.03
Total volatile fatty acids (mmol/L)	89.47	104.06	85.06	94.07	84.86	2.77	0.10

Within rows, means with different letter superscripts (^a,b^) are significantly different (*p* < 0.05).

## Data Availability

The raw sequencing reads were deposited into the NCBI Sequence Read Archive (SRA) data: PRJNA1076690.
